# A mini-invasive surgical technique for Carlevale IOL implantation: case series study and description of concomitant surgery

**DOI:** 10.1007/s00417-023-06217-8

**Published:** 2023-08-30

**Authors:** Carla Danese, Francesco Di Bin, Paolo Lanzetta

**Affiliations:** 1 Department of Medicine – Ophthalmology, University of Udine, Udine, Italy; 2 Ophthalmology Department, AP-HP, Lariboisière Hospital, Université Paris Cité, Paris, France; 3 Istituto Europeo di Microchirurgia Oculare – IEMO, Udine-, Milan, Italy

**Keywords:** Secondary IOL implantation, Carlevale IOL, IOL luxation, IOL opacification, Scleral fixation

## Abstract

**Purpose:**

To examine the feasibility and outcomes of a modified technique for the implantation of scleral fixated Carlevale intraocular lens (IOL) (I71 FIL SSF. Soleko IOL Division, Pontecorvo, Italy), and to analyze the occurrence of adverse events.

**Methods:**

This is a retrospective observational study conducted revising patients charts from 2018 to 2023. Thirty-five eyes of 33 patients were included. Patients requiring IOL explantation had either IOL dislocation or opacification. The implantation of the Carlevale IOL was performed with the subconjunctival positioning of the anchors without any scleral flap. All maneuvers were performed transconjunctivally. The anatomical outcomes considered were IOL positioning, and the absence of postoperative complications. The functional outcomes analyzed were best correctedvisual acuity (BCVA) and refraction.

**Results:**

In all the cases, the IOL was well positioned and centered postoperatively. No cases of conjunctival erosion were recorded. The best corrected visual acuity (BCVA) was 0.9±0.6 logMar (mean±standard deviation) preoperatively and 0.5±0.5 logMar (mean±standard deviation) postoperatively. The mean preoperative spherical equivalent was +6.8±7.7 dioptres, while postoperatively it was -1.1±1.6 dioptres. The most frequent procedure associated to secondary IOL implantation was posterior vitrectomy (25 eyes, 71.4%), which was performed with 25-gauge transconjunctival cannulas in the ciliary sulcus. The follow-up period was 24.5±16.9 months (mean±standard deviation).

**Conclusion:**

The described mini-invasive technique for Carlevale IOL implantation is safe and effective. It can be recommended either as a stand-alone operation or associated to concurrent surgical procedures.

**Supplementary Information:**

The online version contains supplementary material available at 10.1007/s00417-023-06217-8.


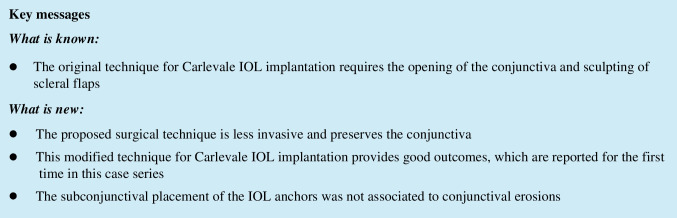


## Introduction

Secondary intraocular lens (IOL) implantation is the preferable surgical procedure in cases of secondary aphakia, IOL dislocation, or opacification [1]. In these eyes, capsular support is often inadequate. During the years, several different surgical techniques have been described in order to achieve good results with minimally invasive techniques. However, no single technique has gained advantage over the others. Advantages and disadvantages vary among all of them. Anterior chamber IOLs include iris suture of a posterior chamber IOL and iris-claw IOLs. Scleral fixation is the most recently developed technique for secondary IOL implantation. A 10-0 polypropylene suture can be used to suture the haptics of the IOL through the ciliary sulcus or, less commonly, through the pars plana. Sutureless scleral fixation has also been described, mainly using three-piece posterior chamber IOLs, such as in the Scharioth technique, with fixation of the haptics into a limbus-parallel tunnel [2, 3].

A novel IOL has been specifically designed for sutureless scleral fixation. The Carlevale lens (I71 FIL SSF. Soleko IOL Division, Pontecorvo, Italy) is a single-piece hydrophilic acrylic IOL, with closed haptics and two protruding T-shaped anchors that allow fixation on the sclera without the need for sutures. More recently, a newer version of the Carlevale IOL has been proposed which has hydrophilic/hydrophobic properties (Carlevale IOL High-Tech. Md-tech, Casoria, Italy). Both types are CE-approved medical devices. The originally described technique requires exposure of the sclera and sculpting two partial thickness scleral lamellas at 0° and 180°. Two sclerotomies are performed through the scleral lamellas. The two T-shaped anchors are externalized through the sclerotomies and placed underneath the scleral lamellas, with the so-called handshake technique, using jaw forceps. The T-shaped anchors, the closed shape of the anchors, the four points of scleral counter-pressure, and the large diameter of the haptic limit tilting and minimize iris chafing [4]. The procedure appears to be safe without significant postoperative complications although there may be conjunctival scarring [5]. Also, sculpting scleral lamellas is time consuming and may be associated with bleeding. Transient clouding of the Carlevale lens due to thermic shock, spontaneously resolving after some hours, has also been reported [5].

Some authors have described modifications of the original implantation technique and the possibility to associate other complementary surgical procedures such as penetrating keratoplasty and Descemet stripping automated endothelial keratoplasty (DSAEK) in eyes with aphakia and corneal failure [6, 7].

We have previously described posterior vitrectomy and Carlevale IOL implantation performed in three cases by placing one cannula into the pars plana and two cannulas into the ciliary sulcus. After completion of vitrectomy, the IOL was implanted with the two anchors externalized and placed beneath the conjunctiva [8].

Hereby, we describe a larger series treated with a mini-invasive technique with a transconjunctival approach in the absence of both opening of the conjunctiva and sculpting the scleral flaps. The procedure was performed either isolated or associated with other surgical procedures including vitrectomy.

## Methods

This is a retrospective case series study conducted revising the clinical charts of patients who underwent Carlevale IOL (I71 FIL SSF. Soleko IOL Division, Pontecorvo, Italy) implantation with the mentioned technique between 2018 and 2023. The study population is composed of 35 eyes of 33 patients. All patients have been operated by the same surgeon (PL) at the Department of Medicine – Ophthalmology of the University of Udine, Udine, Italy. Each patient was examined preoperatively including best corrected visual acuity (BCVA), intraocular pressure (IOP) measurement, slit-lamp examination, optical coherence tomography (OCT) of the macular region, optical or ultrasound biometry. Patients were examined three hours after surgical intervention, the day after, and within one week. Thereafter, the frequency of visits was set according to the clinical situation. For each patient, information regarding BCVA and IOP have been recorded, as well as the ophthalmoscopic findings. Concomitant ocular conditions have also been recorded. The baseline clinical characteristics for each eye are detailed in Table 1. Concomitant ocular conditions limiting the potential improvement of BCVA are also reported. Statistical analysis was performed calculating the mean and standard deviation values for BCVA, spherical equivalent, and astigmatism.

**Table 1:** Detailed information on each eye included in the study

N.	Reason for intervention	Additional procedures	Follow-up	Concurrent conditions	Preop BCVA (logMAR)	Postop BCVA (logMAR)
1	IOL dislocation	25 gauge vitrectomy	56 months	Glaucoma	0.2	0.1
2	IOL dislocation	25 gauge vitrectomy	52 months	Previous acute angle closure glaucoma Macular lipofuscin	0.7	0.5
3	IOL opacification	25 gauge vitrectomy	48 months	None	0.2	0
4	IOL opacification	None	47 months	None	0.2	0
5	IOL opacification	None	47 months	Previous vitrectomy and scleral buckling for retinal detachment	0.4	0.1
6	IOL dislocation	None	46 months	None	0.5	0.1
7	IOL dislocation	25 gauge vitrectomy	42 months	Sarcoidosis Previous optic neuritis	1.8	1.5
8	IOL dislocation	25 gauge vitrectomy	42 months	Amblyopia	0.4	0.3
9	Secondary aphakia and nuclear fragments into the vitreous chamber	25 gauge vitrectomy	39 months	Irvine Gass syndrome	1.0	0.4
10	IOL dislocation	25 gauge vitrectomy	39 months	None	0.7	0.1
11	IOL dislocation	25 gauge vitrectomy	36 months	Glaucoma	0.7	0.3
12	IOL dislocation	25 gauge vitrectomy	34 months	None	1.0	0.1
13	IOL dislocation	25 gauge vitrectomy	34 months	Pathologic myopia Previous myopic choroidal neovascularization	1.8	1.3
14	IOL dislocation	25 gauge vitrectomy	34 months	Glaucoma	1.3	0.2
15	IOL dislocation	25 gauge vitrectomy	34 months	Irvine Gass syndrome	0.3	1.0
16	IOL dislocation	None	31 months	Retinal pigment epithelium distrophy	0.4	0.4
17	IOL dislocation	25 gauge vitrectomy	24 months	None	0.2	0.1
18	IOL dislocation	None	23 months	Epiretinal membrane	1.3	0.2
19	IOL dislocation	25 gauge vitrectomy	21 months	Previous scleral buckling for retinal detachment	0.4	0.4
20	Post traumatic aphakia and retinal detachment	25 gauge vitrectomy	19 months	Previous trauma Retinal detachment	1.8	1.8
21	IOL dislocation and corneal decompensation	DSAEK	14 months	Irvine Gass syndrome	1.8	1.3
22	IOL dislocation	25 gauge vitrectomy	12 months	Glaucoma	1.8	0.5
23	IOL dislocation	25 gauge vitrectomy	12 months	Macular hole	1.3	0.5
24	IOL dislocation	25 gauge vitrectomy	11 months	Geographic atrophy	0.5	0.5
25	Traumatic cataract with dehiscence of the zonular fibers	25 gauge vitrectomy	11 months	Previous acute angle closure glaucoma Epiretinal membrane	0.6	0.1
26	IOL dislocation and corneal decompensation	Penetrating keratoplasty	10 months	Previous uveitis and retinal vasculitis	1.8	1.8
27	IOL opacification	25 gauge vitrectomy	8 months	None	0.3	0.1
28	IOL dislocation	25 gauge vitrectomy	7 months	Pathologic myopia Previous myopic choroidal neovascularization	1.3	0.8
29	IOL opacification	25 gauge vitrectomy	6 months	None	0.3	0
30	IOL dislocation and decompensated glaucoma	Ex-Press shunt implantation	6 months	Age-related macular degeneration and choroidal neovascularization	1.8	1.3
31	Secondary aphakia and nuclear fragments into the vitreous chamber	25 gauge vitrectomy	4 months	Geographic atrophy	1.3	1.0
32	IOL opacification	None	3 months	Previous retinal detachment Primary open angle glaucoma	0.6	0.2
33	IOL dislocation and corneal decompensation	Penetrating keratoplasty	3 months	Previous uveitis and retinal vasculitis	2.3	1.0
34	IOL dislocation	25 gauge vitrectomy	2 months	None	0.5	0
35	IOL dislocation	25 gauge vitrectomy	1 month	Intermediate age-related macular degeneration	0.5	0.4

*BCVA* best corrected visual acuity, *IOL* intraocular lens, *DSAEK* Descemet stripping automated endothelial keratoplasty

## Surgical technique

**Fig. 1:**
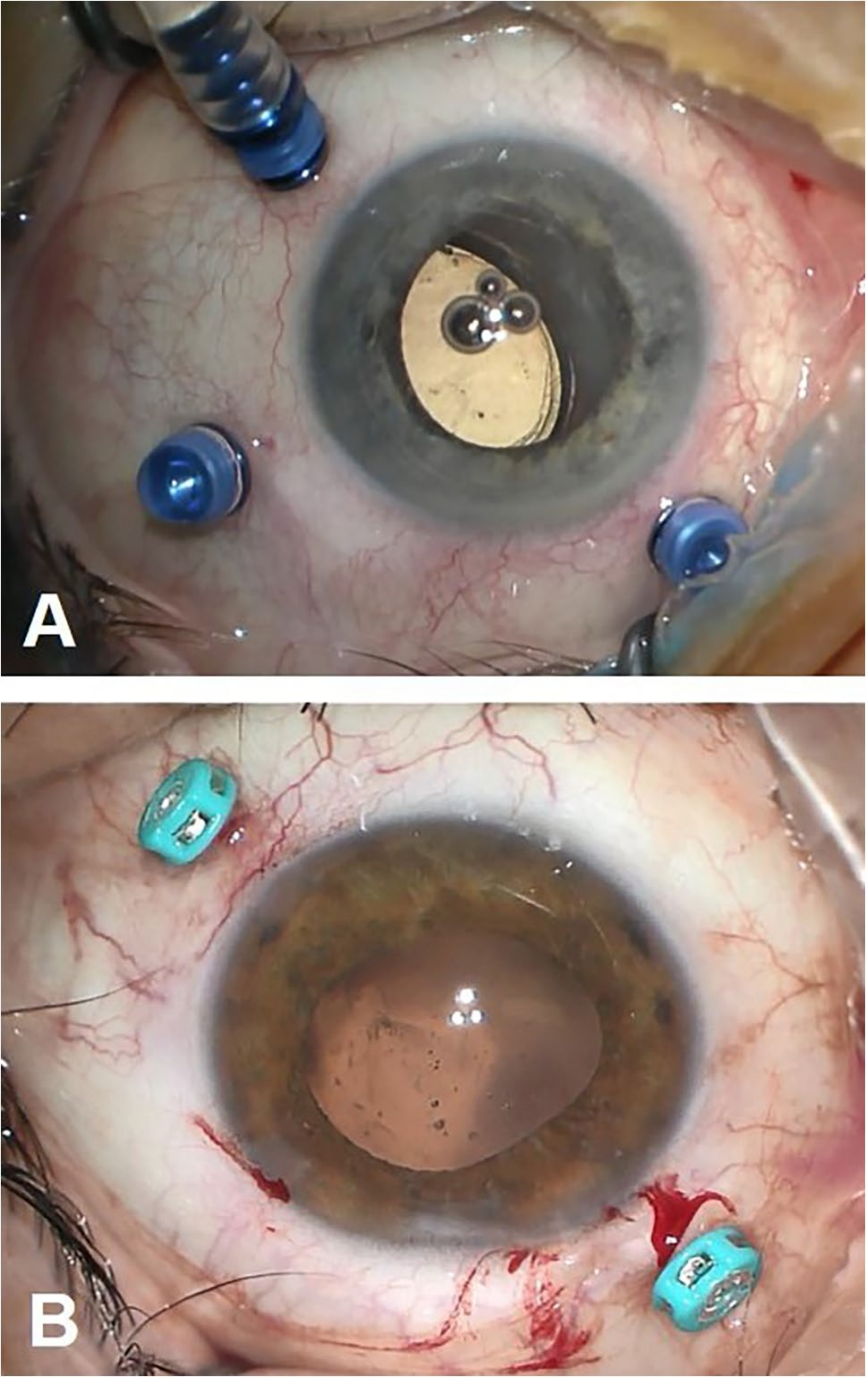
**A** Position of the cannulas in a case requiring associated vitrectomy and dislocated IOL removal. This is a left eye seen from surgeon position. The infero-temporal and supero-nasal cannulas are in the ciliary sulcus, while the supero-temporal cannula is in the pars plana. **B** Position of the cannulas in a case requiring IOL removal and Carlevale IOL implantation. This is also a left eye seen from surgeon position. The infero-temporal and supero-nasal cannulas are positioned in the ciliary sulcus

**Fig. 2:**
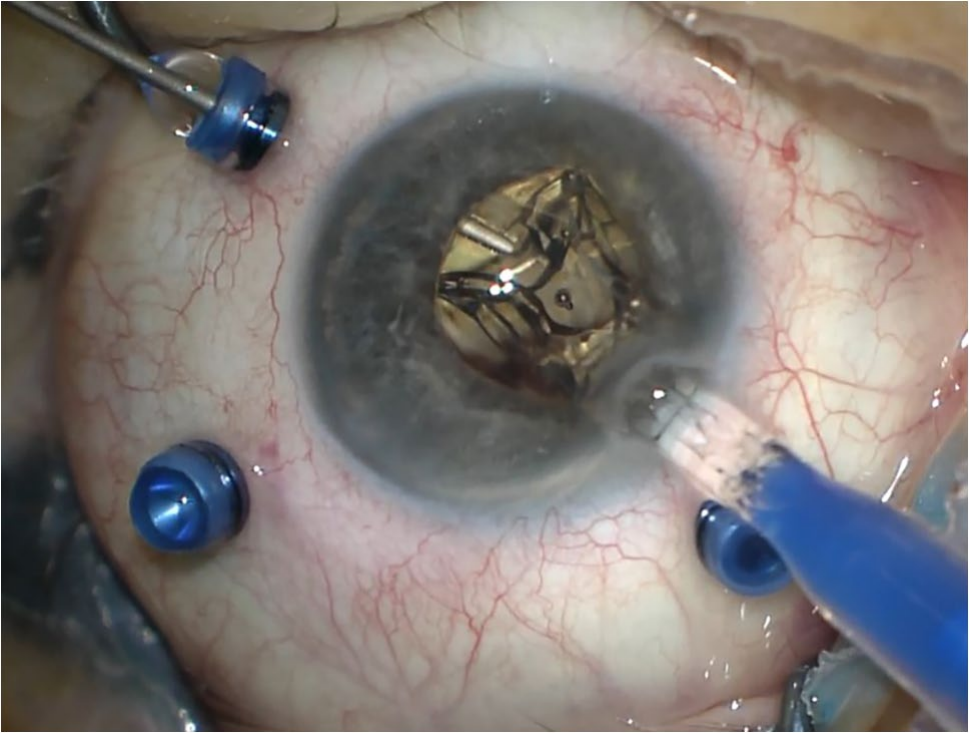
The Carlevale lens is injected through the corneal incision, while one of the anchors is grasped with a jaw forceps

**Fig. 3:**
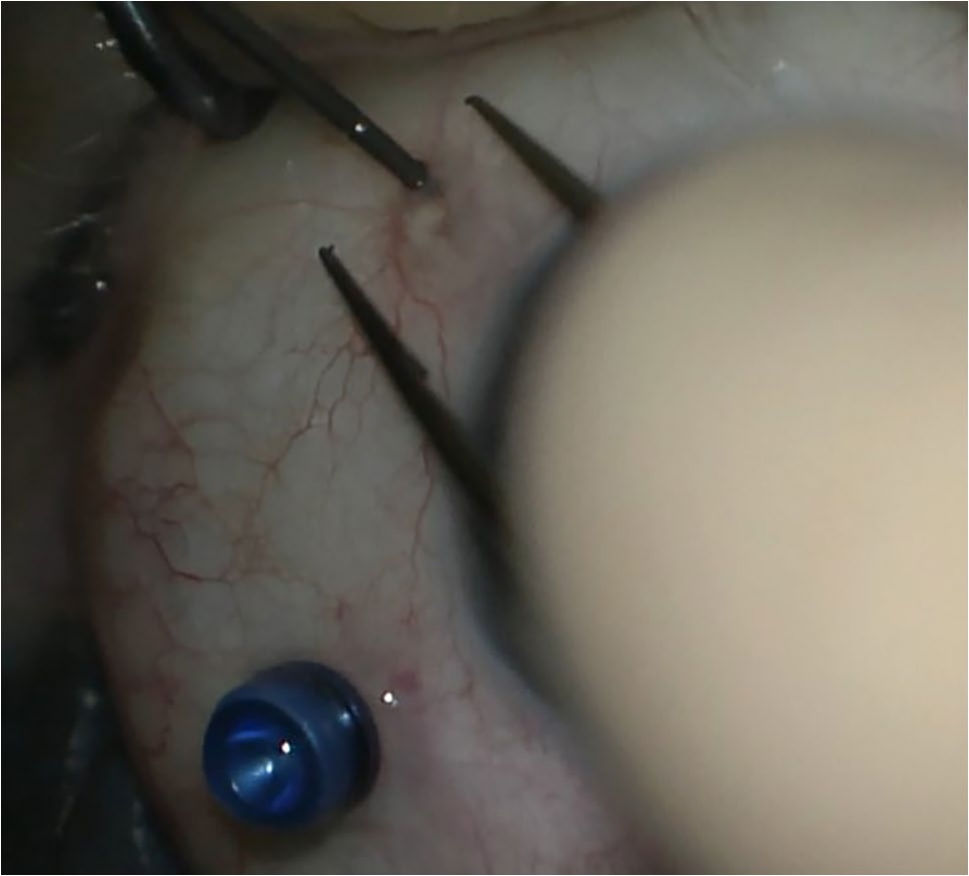
The first anchor is externalized and the cannula is simultaneously removed

**Fig. 4:**
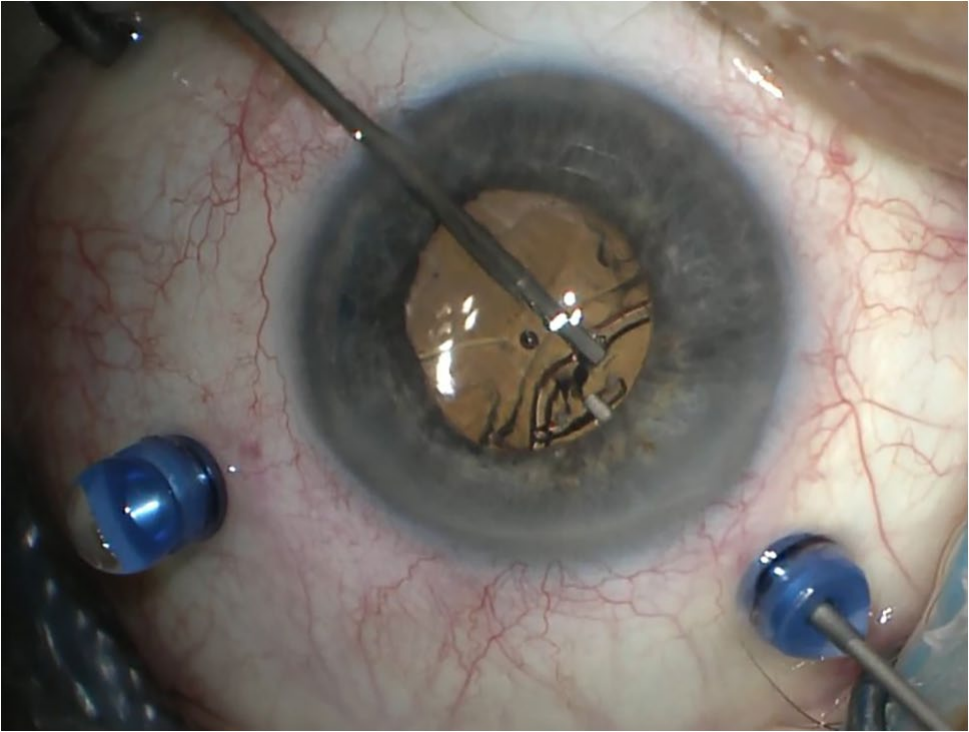
The second anchor is grasped with the handshake technique, using two jaw forceps

**Fig. 5:**
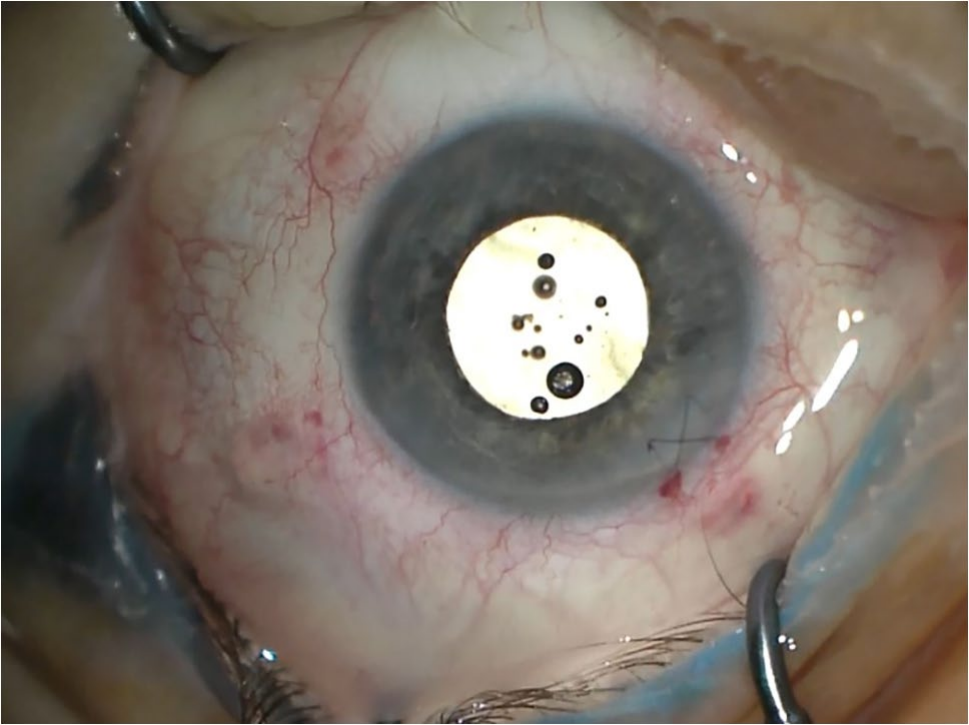
Immediate postoperative result. The lens is centered and the anchors are well positioned beneath the conjunctiva

In the case of IOL implantation without additional posterior segment maneuvers, after the conjunctiva was displaced, two 25-gauge cannulas were placed in the ciliary sulcus, 1.5-2 mm posterior to the limbus, either at 0° and 180° or supero-nasal and infero-temporal. Two corneal incisions along the same axis were performed. In the case of associated vitrectomy, the cannulas were placed in the usual position, supero-nasal, supero-temporal, and infero-temporal with two of them in the ciliary sulcus and one in the pars plana (Fig. 1). Two corneal incisions were placed in the supero-nasal/infero-temporal axis. In all cases, the Carlevale IOL was injected through one of the corneal incisions. During the injection, one of the anchors was grasped with a jaw forceps inserted through one of the cannulas (Fig. 2). The anchor was subsequently externalized and placed beneath the conjunctiva, while the cannula was simultaneously removed (Fig. 3). The second anchor was grasped using two jaw forceps with the so-called handshake technique, and it was also externalized through the cannula, which was simultaneously removed (Fig. 4). No scleral flap was done. An adequate conjunctival displacement was obtained in order to have full sub-conjunctival positioning of the anchors (Fig. 5). When needed, during posterior vitrectomy the displaced IOL and capsular bag, or nuclear fragments, were removed. In order to protect the retina from accidental damage, perfluorocarbon liquids were frequently used.

In Supplementary Video 1, we present the described surgical technique of Carlevale IOL implantation associated to posterior vitrectomy.

## Results

The main surgical indication of the present case series was dislocation of IOL and capsular bag into the vitreous chamber (25 eyes, 71.4%). IOL opacification was reported in six eyes (17.1%). One patient suffered from post-traumatic aphakia and rhegmatogenous retinal detachment. Another patient underwent cataract surgery in another center, complicated by capsular bag rupture and dislocation of nuclear fragments into the vitreous chamber. One patient had a past medical history of contusive trauma, leading to cataract with extensive dehiscence of the zonular fibers and vitreous prolapse into the anterior chamber. In the majority of the cases (25, 71.4%), Carlevale lens implantation was associated with posterior vitrectomy. Depending on the material of the IOL to be explanted, it was either folded or cut and removed through the corneal incision which was also used for implanting the Carlevale lens. In one case, a silicone IOL was explanted through a larger sclero-corneal tunnel due to difficulties in either folding or sawing it into the anterior chamber. In one eye, DSAEK due to corneal decompensation in Fuchs’ dystrophy was performed together with the removal of a dislocated IOL. In two other cases, penetrating keratoplasty for corneal decompensation was associated to three port vitrectomy and Carlevale IOL implantation. The procedure was performed using the Eckardt temporary keratoprosthesis in order to allow improved visualization during vitrectomy. Another patient was aphakic with no capsular bag support as the result of previous globe laceration and secondary rhegmatogenous retinal detachment. Posterior vitrectomy, Carlevale IOL implantation, and silicone oil endotamponade were performed. In one eye suffering from glaucoma, implantation of Ex-press shunt was also carried out.

The follow-up period was 24.5±16.9 months (mean±standard deviation). BCVA was 0.9±0.6 logMar (mean±standard deviation) preoperatively and 0.5±0.5 logMar (mean±standard deviation) postoperatively. Mean (± standard deviation) preoperative spherical equivalent was 6.8±7.7 diopters, while postoperatively it was -1.1±1.6 diopters. Mean (± standard deviation) preoperative astigmatism was -0.1±0.8 diopters, while postoperatively it was -0.9±1.9 diopters. The values of spherical equivalent and of astigmatism were obtained with objective refraction, using an auto-refractometer (Topcon TRK-2P, Tokyo, Japan). The refractive target was reached in all cases, with no cases of refractive shift. All the IOLs were centered postoperatively. No cases of IOL tilting evaluated at the slit-lamp with a dilated pupil were reported. IOP elevation or hypotony were not seen and in no cases the positioning of the cannulas in the ciliary sulcus led to intraoperative or postoperative bleeding. In a single case, one of the anchors of the IOL partially ruptured at the end of the operation leaving an irregular surface under the conjunctiva and causing a conjunctival granuloma some months later. The granuloma was excised and the damaged anchor was covered with a scleral patch. Overall, no cases of conjunctival erosions were noted. No endophthalmitis events were reported.

## Discussion

Secondary IOL implantation is a useful procedure in several conditions. IOL and capsular bag dislocation are well described in literature, especially in patients with zonular dehiscence such as those with pseudoexfoliation syndrome [9]. Secondary aphakia in the absence of proper capsular support as consequence of eye trauma is another relevant indication [10]. Moreover, some IOLs are prone to develop opacifications in the postoperative period [11]. Therefore, several techniques have been developed in order to perform secondary IOL implantation. It is also well accepted that surgery should be less invasive as possible. The Carlevale lens represents a good solution, providing good IOL stability in the absence of a capsular bag support. The described postoperative outcomes are usually satisfying [4]. The present study describes a modified technique for Carlevale IOL implantation, which has been developed in order to reduce invasivity. This is performed without opening the conjunctiva and avoiding sculpting the scleral flaps and by placing the anchors through the ciliary sulcus directly into the subconjunctival space. By displacing the conjunctiva at the time of trocar entry, once the anchors are extruded through the sclera they will be covered by intact conjunctiva. When vitrectomy is needed, the cannulas positioned in the ciliary sulcus are also used for the insertion of the vitrectomy instruments [8]. Importantly, the jaw forceps need to be manually curved by the surgeon prior to IOL implantation in order to favor the grasping of the anchors. The implantation of the Carlevale IOL has been a useful tool also when treating eyes with a history of trauma. The proposed Carlevale implantation technique was also used without modification in case of other concurrent procedures such as vitrectomy, and cornea or glaucoma surgery. BCVA improved in all the patients enrolled in the present study. The refractive target was reached in all cases and the magnitude of improvement was correlated to the underlying preoperative conditions. We did not observe any case of hypotony due to leakage through the scleral incisions as the haptics of the IOL fully fill the 25-gauge sclerotomies.

The most relevant postoperative outcomes are adequate IOL positioning and the absence of conjunctival erosions, i.e., the loss of conjunctival tissue with exposure of the anchors. All the IOLs remained well centered without tilting – i.e., the undesired rotation of the IOL on the axis of insertion – throughout the whole follow up period. Centration and tilting of the IOL were evaluated at the slit-lamp before and after pupil dilation. As already described in literature, the specific design of the IOL and the large dimensions of the optic plate make it especially stable and less prone to tilting [4, 12]. Despite these satisfactory results, a precise evaluation of IOL tilting can only be performed using anterior segment OCT, a Scheimpflug camera, or ultrasound biomicroscopy, as described in literature [13]. These instruments could also allow a more precise analysis of preoperative and postoperative astigmatism. The anchors maintained the correct position underneath the conjunctiva and there has been no evidence of conjunctival erosions. It is likely that the hydrophilic material of the Carlevale lens is soft enough to allow subconjunctival placement of the anchors without any major damage to the overlying conjunctiva. In the present case series, it was tolerated without any inflammation or erosion. We may also assume that the conjunctival granuloma shown by one of the patients was caused by the irregular edge of a broken plug, since it occurred in that single case. At slit-lamp examination, the position of the IOL into the ciliary sulcus was considered satisfactory. However, only OCT or ultrasound biomicroscopy can precisely determine the exact position of the IOL. The possibility of performing concomitant surgery in the same operating time is also interesting, since these are often complex eyes with more than one condition to be addressed. As mentioned, the cannulas into the ciliary sulcus are useful to perform posterior vitrectomy, which is often needed due to dislocation of the IOL-capsular bag complex into the vitreous chamber or to secondary aphakia with vitreous prolapse into the anterior chamber [8]. Corneal surgery, either lamellar or full thickness, has also been successfully associated with Carlevale IOL implantation during the same operating time.

In conclusion, we believe that this modified technique for Carlevale IOL implantation is safe and effective. Its main advantage in relation to the traditional technique is its reduced invasivity. This technique can be successfully performed with good results in cases requiring secondary IOL implantation, either isolated or associated with concomitant surgery. Further studies are encouraged to evaluate the results of this technique on larger cohorts of patients with a longer follow-up.

### Supplementary information

Supplementary file 1 **Caption of Supplementary Video 1** This video shows removal of dislocated IOL from the vitreous chamber, 25 gauge vitrectomy, and Carlevale IOL implantation. The surgical technique is described in the main text. This is a right eye seen from the surgeon position. (MP4 317191 kb)
